# Midstance instant identification methods in sprinting: comparative assessment and biomechanical implications in able-bodied athletes and athletes with lower limb-amputation

**DOI:** 10.3389/fbioe.2026.1882187

**Published:** 2026-09-04

**Authors:** Paola Righetto, Sara Faggian, Giuseppe Marcolin, Gian Luca Migliore, Andrea Giovanni Cutti, Nicola Petrone, Roberto Di Marco

**Affiliations:** 1 Department of Industrial Engineering, University of Padova, Padova, Italy; 2 Department of Biomedical Sciences, University of Padova, Padova, Italy; 3 Centro Protesi, INAIL, Budrio, Italy

**Keywords:** kinematics, kinetics, midstance, prostheses, sprinters

## Abstract

**Introduction:**

Midstance is a key instant in running, marking the transition from braking phase to propulsion phase. Despite its significance for stability and force transmission, a standard identification method of midstance instant is lacking, leading to inconsistent data interpretations. This study aimed to systematically compare different midstance instant identification methods and to highlight how they affect trunk and lower-limb kinematics in both able-bodied sprinters and sprinters with lower limb amputation.

**Methods:**

22 able-bodied athletes, 4 athletes with unilateral transtibial and 4 athletes with unilateral transfemoral amputation performed two maximal 60-m sprints on an instrumented track. Kinematic and kinetic data were recorded using 10 infrared cameras and 9 embedded force platforms. Seven methods were considered, including kinematic and kinetic approaches. Sagittal absolute angles of trunk, thigh, and shank segments, as well as the hip, knee and ankle joints were extracted at the instants identified by each method. Both temporal differences and corresponding body postures at the detected instants were evaluated across methods and against the theoretical midpoint of stance (50%). Differences were assessed using either one-way repeated measures ANOVA or Friedman tests, depending on data normality, followed by Bonferroni post-hoc comparisons when appropriate.

**Results:**

Instants identified as midstance consistently preceded the temporal midpoint of the stance phase across methods considering both able-bodied and athletes with lower-limb amputation. Kinematic methods showed limited consistency, identifying different instants within the stance phase. Their agreement with kinetic-based methods was only partial and depended on both the identification method and the athlete group. Consequently, midstance identified with different methods reflected distinct body postures within the stance phase. This resulted in significant variations in segment and joint angles, potentially leading to different biomechanical interpretations of sprinting.

**Discussion:**

Since the present findings suggest that method performance may differ across able-bodied athletes, athletes with transtibial amputation, and athletes with transfemoral amputation, researchers and practitioners should carefully consider the choice of method, clearly disclose it and ensure its consistent application.

## 1 Introduction

Running is a cyclical movement pattern composed of alternating stance and swing phases (Kapri et al., 2021). Within the stance phase, the midstance instant represents a key event, marking the transition from the braking phase to the propulsion phase. At this moment, the body’s center of mass passes over the supporting limb through a reduced base of support, making it crucial for maintaining the athlete’s stability (Silva and Stergiou, 2020).

Midstance was initially defined for walking as a phase (Perry and Burnfield, 2010) and later extended to running as the instant marking the transition between the braking and propulsion phase (Ounpuu, 1990; Thordarson, 1997; Dugan and Krishna, 2005; Richards, 2008; Inostroza-Ríos et al., 2025). However, this definition was never supported by experimental data. Midstance in running has also been defined as the theoretical midpoint of the stance phase (Blickhan, 1989). In applied settings, coaches frequently rely on visual identification of midstance using 2D video analysis, often referred to as the “coach’s eye” (Lath et al., 2021). One commonly adopted criterion is the instant at which the longitudinal axis of the shank contralateral to the support limb is parallel to the ground. Although not formally standardized in scientific literature, shank angle has been used in 2D running assessment to evaluate the loading response and as a practical surrogate for identifying overstriding (Souza, 2016). Alternatively, midstance is sometimes identified as the instant when the greater trochanter (GT) of the supporting limb is vertically aligned with the lateral malleolus (LM), reflecting a key position described in the Pose® Method of running (Romanov 2002; Romanov and Fletcher, 2007). This method underpins a widely used field-based coaching program (Wei et al., 2021) designed to modify running technique to improve performance and reduce injury risk (Arendse et al., 2004; Fletcher et al., 2008). The method focuses on evaluating a single “pose” during stance, characterized by the vertical alignment of the acromion, GT, and LM, also referred to as “S-like” body posture (Arendse et al., 2004). A similar configuration is employed within the Sprint Mechanics Assessment Score, a qualitative field-based screening tool. In this protocol, midstance, defined as the instant when the pelvis is positioned over the ankle joint, is critical for identifying the vertical collapse, one of the twelve parameters contributing to the Sprint Mechanics Assessment Score value and, therefore, to the assessment of sprint mechanics (Bramah et al., 2024).

The lack of a shared and context-specific definition of midstance (Inostroza-Ríos et al., 2025) is not limited to able-bodied (AB) runners but also extends to athletes with lower-limb amputation, who compete using running-specific prostheses (RSPs) (Hadj-Moussa et al., 2022). Definitions of midstance in running remain similarly inconsistent for people with transtibial (PTTA) or transfemoral (PTFA) amputation. Some authors identify midstance as the instant corresponding to the peak of vertical ground reaction force, as it typically occurs around the temporal midpoint of stance (Beck et al., 2016). Migliore et al. (2021) defined midstance, for the affected limb (AL) in PTFA, as the instant when the vertical projection of the GT aligns with the prosthetic knee center (K). More generally, prosthetists assess RSP alignment through visual cues derived from clinical experience to ensure stability during the stance phase (Chen et al., 2016).

The lack of consensus on methods for identifying the midstance instant in sprinting, in both able-bodied athletes and athletes with lower-limb amputation, leads to ambiguity in communications among coaches, clinicians, and researchers. Different identification methods may correspond to different instants of the stance phase, thereby resulting in inconsistent analysis of the running technique (Inostroza-Ríos et al., 2025). This variability can limit the effectiveness of corrective feedback in able-bodied athletes and complicate the tuning and alignment of RSP in Paralympic runners. Indeed, midstance identification is crucial for both populations, since it plays a fundamental role for optimizing running technique in able-bodied athletes and, in athletes with lower-limb amputation, for prosthetic tuning and alignment, which in turn affect overall running mechanics.

Therefore, the first aim of this study was to systematically compare the timing of the midstance instant during sprinting as identified by different methods in able-bodied athletes and para-athletes with transtibial and transfemoral amputation. The investigated methods include both approaches derived from the scientific literature and those commonly used in applied practice by coaches and practitioners. As these approaches are employed in real-world settings, their potential differences are of practical relevance for both researchers and practitioners in the field (Inostroza-Ríos et al., 2025).

The second aim of the study was to quantify differences in trunk and lower-limb kinematics associated with midstance instants identified using the different methods. This comparison may provide practical insights for coaches and practitioners, as different definitions of midstance may correspond to different athlete postures, potentially leading to different interpretations of sprinting biomechanics.

## 2 Materials and methods

A cross-sectional study design was adopted to compare different methods for midstance identification. Data acquisition was conducted over an instrumented 60-m lane in a certified indoor track and field facility (Palaindoor, Padova, Italy). This study was approved by the Human Ethical Committee of the Department of Biomedical Sciences of the University of Padova (HEC-DSB/1–2024) and followed the Declaration of Helsinki guidelines. All participants provided written informed consent.

### 2.1 Participants

Inclusion criteria for the participation in the study were: (i) age between 18 and 35 years old; (ii) sprint running athlete in 60-, 100-, or 200-m; (iii) for AB athletes, best 100-m sprint running time up to 2 months prior to data acquisition <12.5 s; (iv) for athletes with lower limb amputation, best 100-m sprint running time up to 2 months prior to data acquisition <15 s. Athletes who experienced musculoskeletal acute injuries at the time of the study were excluded. Sprinters with transtibial and transfemoral unilateral amputation had to compete in track and field categories of either T64 or T63.

The final sample consisted of 22 AB, 4 PTTA, and 4 PTFA sprinters. Demographic and anthropometric characteristics are detailed in Table 1.

**TABLE 1:** Demographic and anthropometric characteristics.

Group	All (females)	Age [y]	Body mass [kg]	Body height [m]	SBRT [s]
AB	22 (4)	22.57 ± 4.37	70.89 ± 9.08	1.77 ± 0.09	11.03 ± 0.42
PTTA	4 (1)	23.04 ± 6.08	67.75 ± 14.86	1.74 ± 0.08	11.80 ± 1.24
PTFA	4 (3)	33.85 ± 8.59	59.50 ± 12.40	1.65 ± 0.10	13.86 ± 0.93

Abbreviations: AB–able-bodied athletes; SBRT–season’s best running time; PTTA–people with transtibial amputation; PTFA–people with transfemoral amputation.

### 2.2 Procedures

Participants performed two maximal 60-m sprints from a standing start. AB wore spiked track shoes, while athletes with amputation used a spiked shoe on the sound limb and a spiked sole on the running prosthetic foot, as specified by the manufacturers. A 6-min rest interval was provided between trials, preceded by a 45-min self-administered warm-up.

**FIGURE 1:**
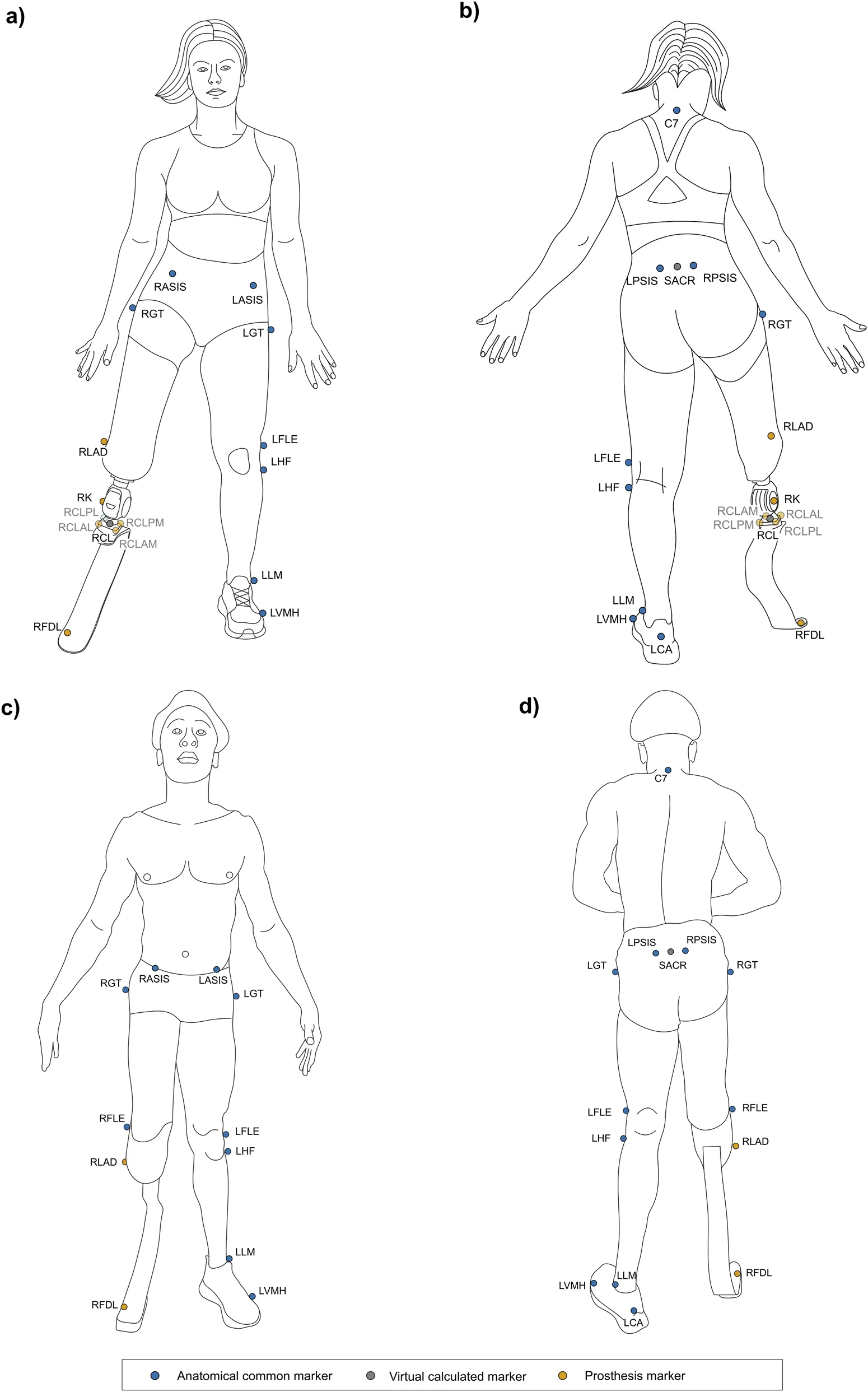
Marker set adopted in the study. Sprinters with transfemoral **(a,b)** and transtibial **(c,d)** prostheses are presented. Anatomical markers are shown in blue, virtual markers in grey, and prosthesis markers in yellow. Only anatomical and virtual markers were used for able‐bodied sprinters. ASIS: anterior-superior iliac spine, C7: 7th cervical vertebra, CA: calcaneus, CL: clamp center, CLAL: anterior lateral screw of the clamp, CLAM: anterior medial screw of the clamp, CLPL: posterior lateral screw of the clamp, CLPM: posterior medial screw of the clamp, FDL: lateral marker of the RSP, 5 cm from its tip, FLE: femur lateral epicondyle, GT: greater trochanter, HF: head of fibula, K: prosthetic knee center, LAD: distally on the lateral axis of the socket, LM: lateral malleolus, PSIS: posterior-superior iliac spine, SACR: sacrum as midpoint between PSIS, VMH: 5th metatarsal distal head. Before each acronym, L: left, R: right.

Data were collected using the “Olympia SmartTrack” system installed at the “Palaindoor Padova”, consisting of: (i) a 13.0 × 7.0 × 3.5 m^3^ portal to carry the (ii) 10-IR camera stereophotogrammetric system (Vicon Valkyrie, Vicon Motion Systems Ltd., Oxford, United Kingdom), and (iii) nine force platforms embedded in the running lane (two BMS400600 and seven BMS600900, AMTI Advanced Mechanical Technology, Inc., Watertown, MA, United States) for ground reaction force acquisition. The instrumented track section was positioned centrally along the running lane, so to capture athletes’ steady state running. Marker trajectories were recorded at 250 Hz to estimate kinematics, and ground reactions were recorded at 1,500 Hz. Following anatomical or mechanical landmarks identification (Di Marco et al., 2025), reflective markers were placed to the participant’s skin or prosthesis (Figure 1) using a double-sided tape and then secured with kinesiology tape (Uchidodo, Japan). Only the sagittal-plane kinematics was considered for the purpose of the present study.

### 2.3 Data analysis

The dependent variables of the study were temporal and kinematic outcomes at midstance, whereas the independent variables were the methods used to identify the midstance instant.

The primary parameter used to compare the different midstance identification methods was the percentage of the stance phase at which midstance instant occurred following the application of each method. In addition, absolute and relative sagittal-plane angles were extracted in accordance with the International Society of Biomechanics recommendations (Wu and Cavanagh, 1995; Wu et al., 2002; 2005), after the marker trajectories were labelled and filtered using a fourth order zero-lag low-pass Butterworth filter with a 10 Hz cut-off frequency. Following a spectral analysis, ground reaction forces (GRFs) were filtered using a fourth order zero-lag low-pass Butterworth filter with a 100 Hz cut-off frequency to remove background noise. Foot-strike (FS) and foot-off (FO) were visually identified from the filtered vertical GRF to ensure accurate event detection, in accordance with established practice in motion capture data analysis (Armand et al., 2024).

The reconstruction of the human body model was done according to 17 markers for the AB athletes, 16 for the PTTA, and 21 for the PTFA, respectively (Di Marco et al., 2025). For AB sprinters, as well as for the unaffected limb (UL) of PTTA and PTFA, 2D body segments were defined as follows: trunk, C7-SACR; thigh, GT–FLE; shank, HF–LM; and foot, CA–VMH. For the affected limb in PTTA, the shank segment was defined as FLE–LAD. In PTFA, the affected limb segments were defined as: thigh, GT–LAD; and shank, K–CL. Absolute angles of the trunk, thigh, and shank were defined as the angles between each segment and the global vertical axis. Relative hip, knee, and ankle joint angles were computed for AB limbs and for the unaffected limb of athletes with lower-limb amputation. For the affected limb, the same joint angles were computed excluding the ankle, as it represents a virtual parameter that does not directly describe prosthetic alignment.

**FIGURE 2:**
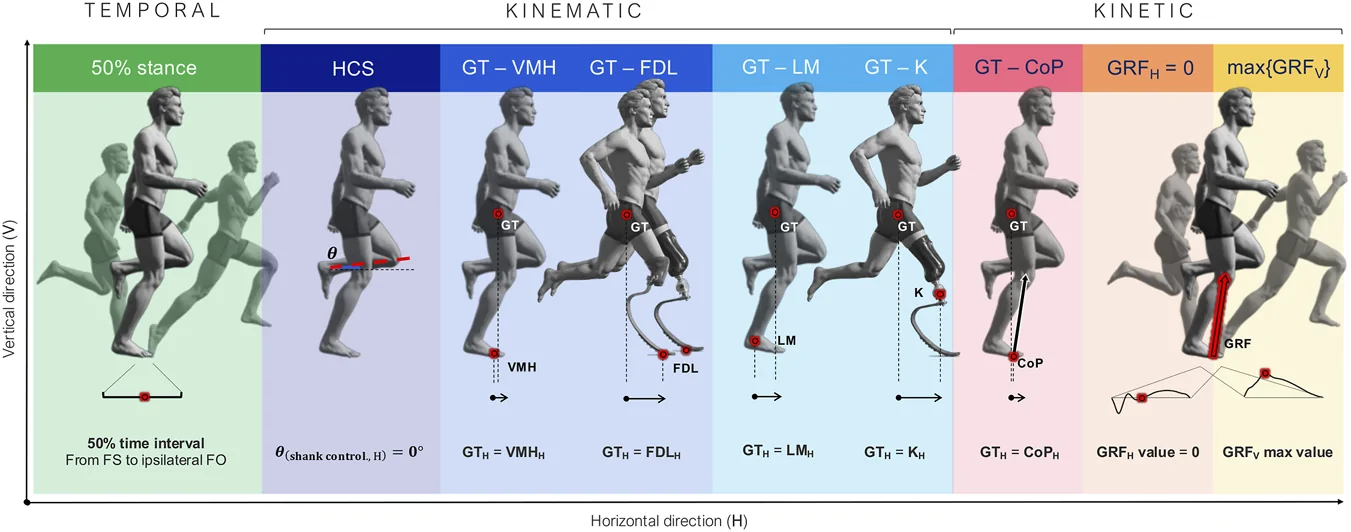
Overview of the methods used to identify the midstance instant in sprinting, with the temporal method highlighted in dark green, the kinematic-based methods highlighted with a gradient of blue, and kinetic-based methods highlighted with a gradient of orange. Abbreviations: CoP–center of pressure; FDL–lateral marker of the RSP, 5 cm from its tip; GRF–ground reaction force; GT–greater trochanter; H–horizontal (i.e., anterior-posterior); HCS–horizontal contralateral shank; K–prosthetic knee center; LM–lateral malleolus; V–vertical; VMH‐5th metatarsal distal head.

Seven methods for identifying midstance instant were compared within AB, PTTA and PTFA groups. These methods were classified into three categories: temporal, including one method, kinematic, including three methods, and kinetic, including three methods (see Table 2; Figure 2). Both temporal discrepancies and body posture at the detected midstance instants were compared across methods, also considering the deviations from the first one (i.e., the theoretical midpoint of the stance phase – 50% stance) and the corresponding body posture.

**TABLE 2:** Overview of the methods used to identify the midstance instant in sprinting, including method category, operational definition, and applicability across groups and limbs.

ID	Method name	Category	Operational definition	AB	PTTA		PTFA	
					UL	AL	UL	AL
1	50% stance	Temporal	Theoretical midpoint of stance phase (50% of FS–FO distance)	✓	✓	✓	✓	✓
2	HCS (horizontal contralateral shank)	Kinematic	Contralateral shank parallel to the global anterior-posterior axis	✓	✓	✓	✗	✓
3	GT – <*foot point*> GT – VMH GT – FDL	Kinematic	Horizontal coordinate of GT equals the horizontal coordinate of VMH	✓	✓	✗	✓	✗
			Horizontal coordinate of GT equals the horizontal coordinate of FDL	✗	✗	✓	✗	✓
4	GT – <*knee/ankle point*> GT – LM GT – K	Kinematic	Horizontal coordinate of GT equals the horizontal coordinate of LM	✓	✓	✗	✓	✗
			Horizontal coordinate of GT equals the horizontal coordinate of K	✗	✗	✗	✗	✓
5	GT – CoP	Kinetic	Horizontal coordinate of GT equals the horizontal coordinate of CoP	✓	✓	✓	✓	✓
6	GRF_H_ = 0	Kinetic	GRF horizontal component equals zero	✓	✓	✓	✓	✓
7	max{GRF_V_}	Kinetic	Peak of the GRF vertical component	✓	✓	✓	✓	✓

Applicability is reported for able-bodied athletes (AB) and athletes with transtibial (PTTA) and transfemoral (PTFA) amputation, considering the unaffected limb (UL) and affected limb (AL). Check marks (✓) indicate applicability of the method, whereas crosses (✗) indicate non-applicability.

The differences in the body landmarks across groups were considered when identifying the midstance methods. Accordingly, the GT–FDL method used for PTTA and PTFA serves as a substitute to the GT–VMH method used for AB athletes. Conversely, the GT–LM method applied in AB athletes does not have a corresponding method for the affected limb of PTTA and PTFA due to the absence of the ankle. Furthermore, the GT–K method was applicable only to the affected limb of PTFA because of the presence of the prosthetic knee, while the HCS method was not applicable to the unaffected side of PTFA. All parameters were averaged among the 2 recorded sprints. The right and left side of AB athletes were averaged, whereas the unaffected and the affected limb of PTTA and PTFA were analyzed separately. The analysis tools were developed with MATLAB R2024b (The MathWorks, Inc., MA, United States).

### 2.4 Statistical analysis

Normality of the data distribution was assessed using the Shapiro-Wilk test. Data are presented as mean ± standard deviation for normally distributed, and as median (1^st^ quartile and 3^rd^ quartile) for non-normally distributed. Accordingly, differences among the seven midstance identification methods were tested using a one-way repeated-measures analysis of variance (ANOVA) for normally distributed outcomes, or the Friedman test for non-normally distributed outcomes. When appropriate, Bonferroni-adjusted *post hoc* comparisons were performed. Effect size was measured as partial eta squared ( $\eta_{p}^{2}$ ) for the main effect and the Cohen’s *d* for *post hoc* comparisons in the ANOVA, whereas the Kendall’s W (W) was considered for the Friedman test. The significance level was set as *p* < 0.05.

To quantify the inter-subject variability for each method, the mean or median absolute deviation (MAD) was calculated for normally distributed and non-normally distributed data, respectively (see Supplementary Material). For comparability across methods, each MAD value was normalized to the lowest within-method MAD, yielding the relative MAD (rMAD), with rMAD ≥1 and larger values indicating larger variability. Moreover, the intraclass correlation coefficients (ICCs) were calculated for the AB group using a two-way random-effects model with absolute agreement and single measures (ICC(2,1)) to assess consistency among methods for each outcome (Shrout and Fleiss, 1979; Koo and Li, 2016). The small sample size did not allow to apply the same analysis to the PTFA and PTTA groups. Statistical analyses were conducted in JASP (0.19.1 University of Amsterdam, Netherlands).

## 3 Results

**FIGURE 3:**
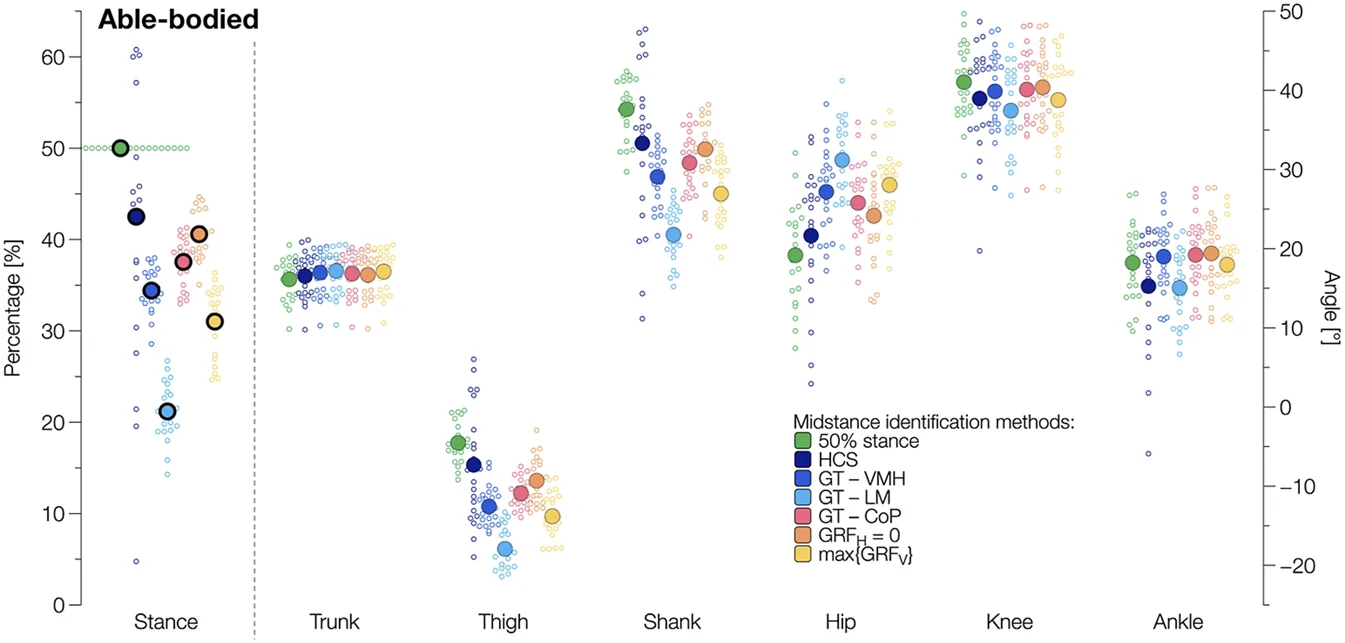
Distribution of kinematic and temporal parameters at midstance in able-bodied sprinters. Data are presented according to the mean and participants’ distribution. Abbreviations: CoP–center of pressure; GRF–ground reaction force; GT–greater trochanter; H–horizontal (i.e., anterior-posterior); HCS–horizontal contralateral shank; LM–lateral malleolus; V–vertical; VMH‐5th metatarsal distal head.

**FIGURE 8:**
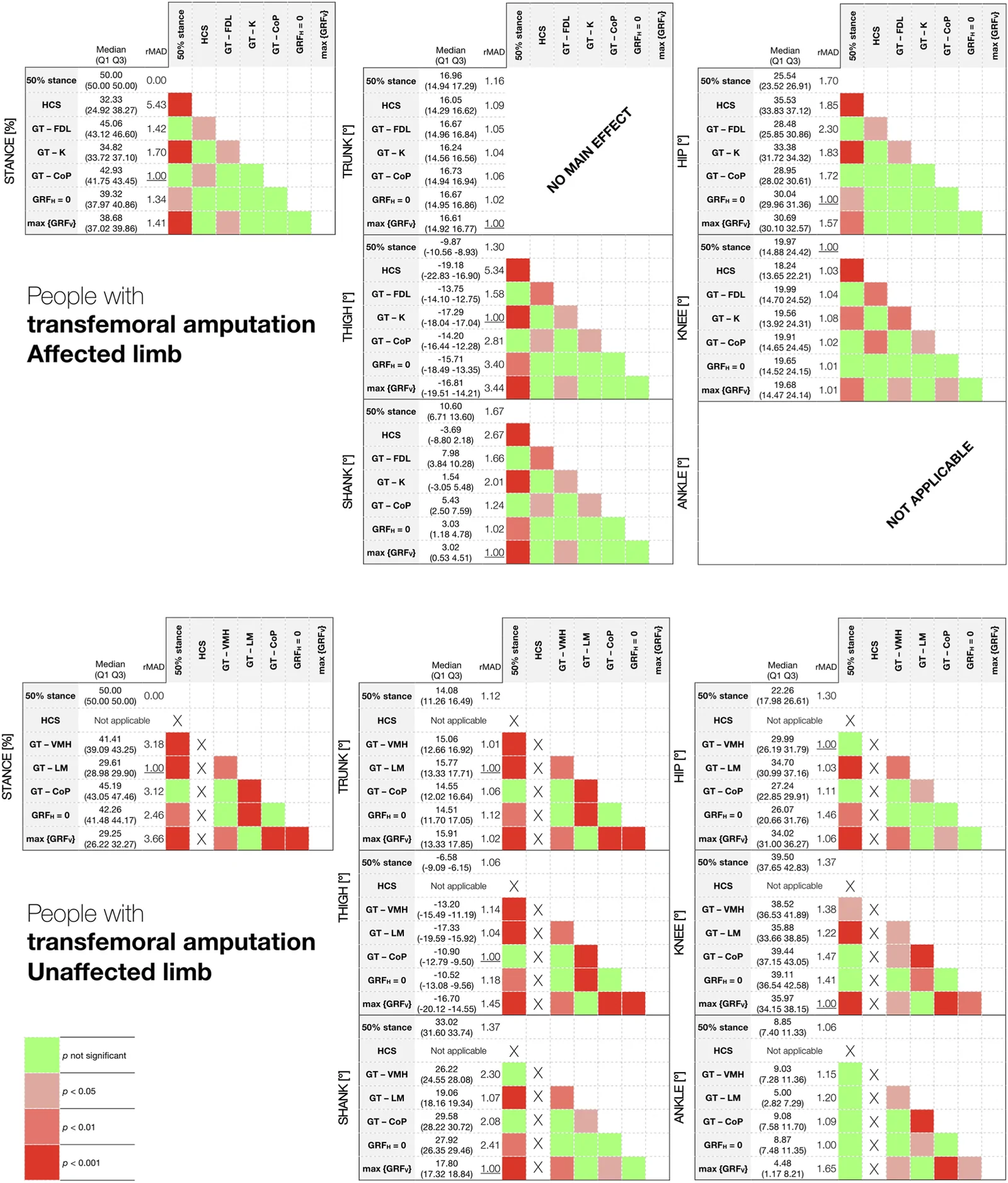
Post-hoc comparison between midstance identification methods in sprinters with transfemoral amputation. Data are reported as median (1st quartile 3rd quartile) and relative median absolute deviation. Comparisons between methods are represented by T-intersections in the tables. Statistical significance is represented by the color scale. Abbreviations: CoP–center of pressure; FDL–lateral marker of the RSP, 5 cm from its tip; GRF–ground reaction force; GT–greater trochanter; H–horizontal (i.e., anterior-posterior); HCS–horizontal contralateral shank; K–knee prosthetic center; LM–lateral malleolus; rMAD–relative median absolute deviation; V–vertical; VMH‐5th metatarsal distal head.

**FIGURE 4:**
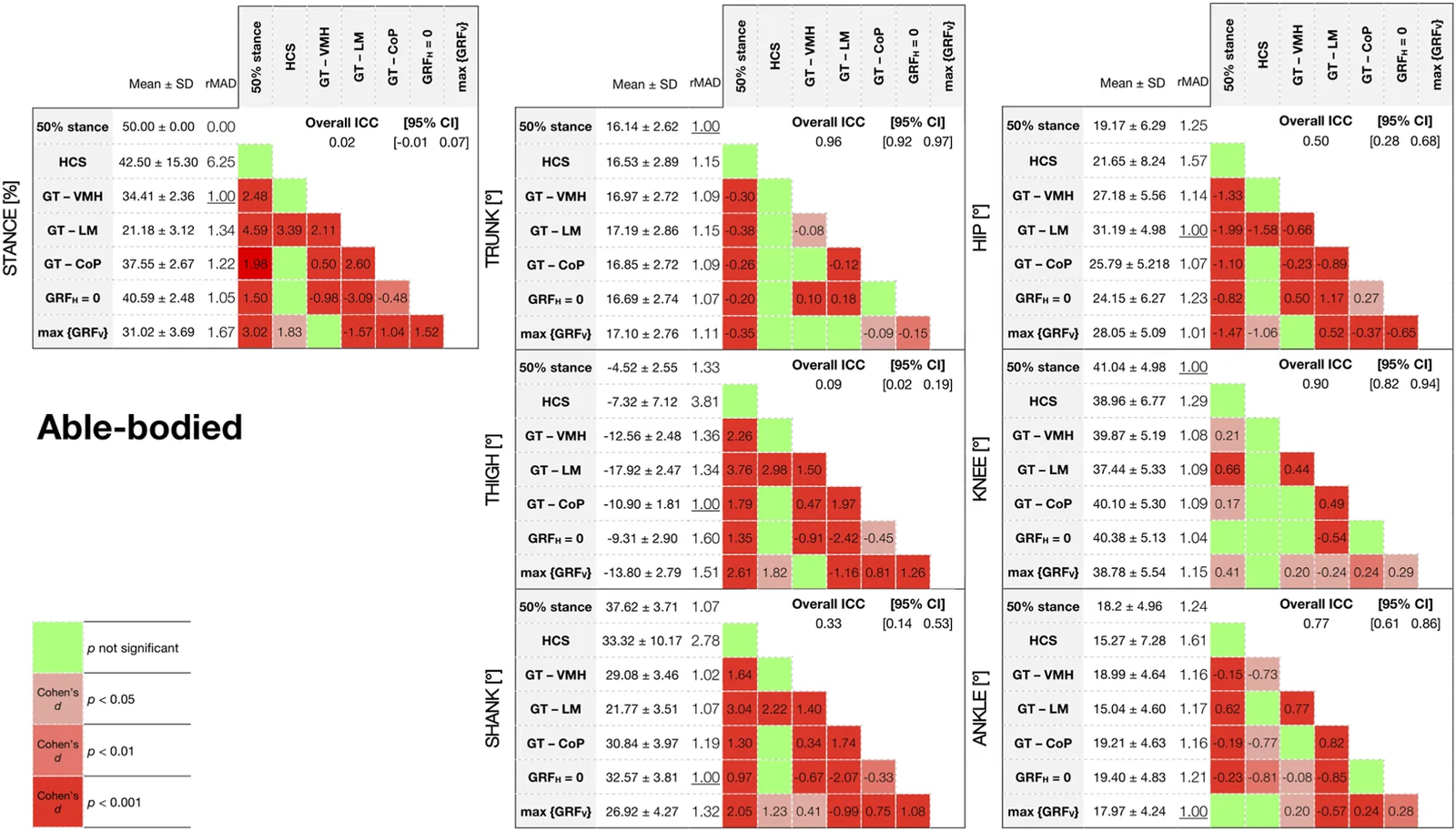
Post-hoc comparison between midstance identification methods in able-bodied sprinters. Data are reported as mean ± standard deviation and relative mean absolute deviation. Comparisons between methods are represented by T-intersections in the tables. Statistical significance is represented by the color scale. Abbreviations: CI–confidence interval; CoP–center of pressure; GRF–ground reaction force; GT–greater trochanter; H–horizontal (i.e., anterior-posterior); HCS–horizontal contralateral shank; ICC–intraclass correlation; LM–lateral malleolus; rMAD–relative mean absolute deviation; V–vertical; VMH‐5th metatarsal distal head.

**FIGURE 5:**
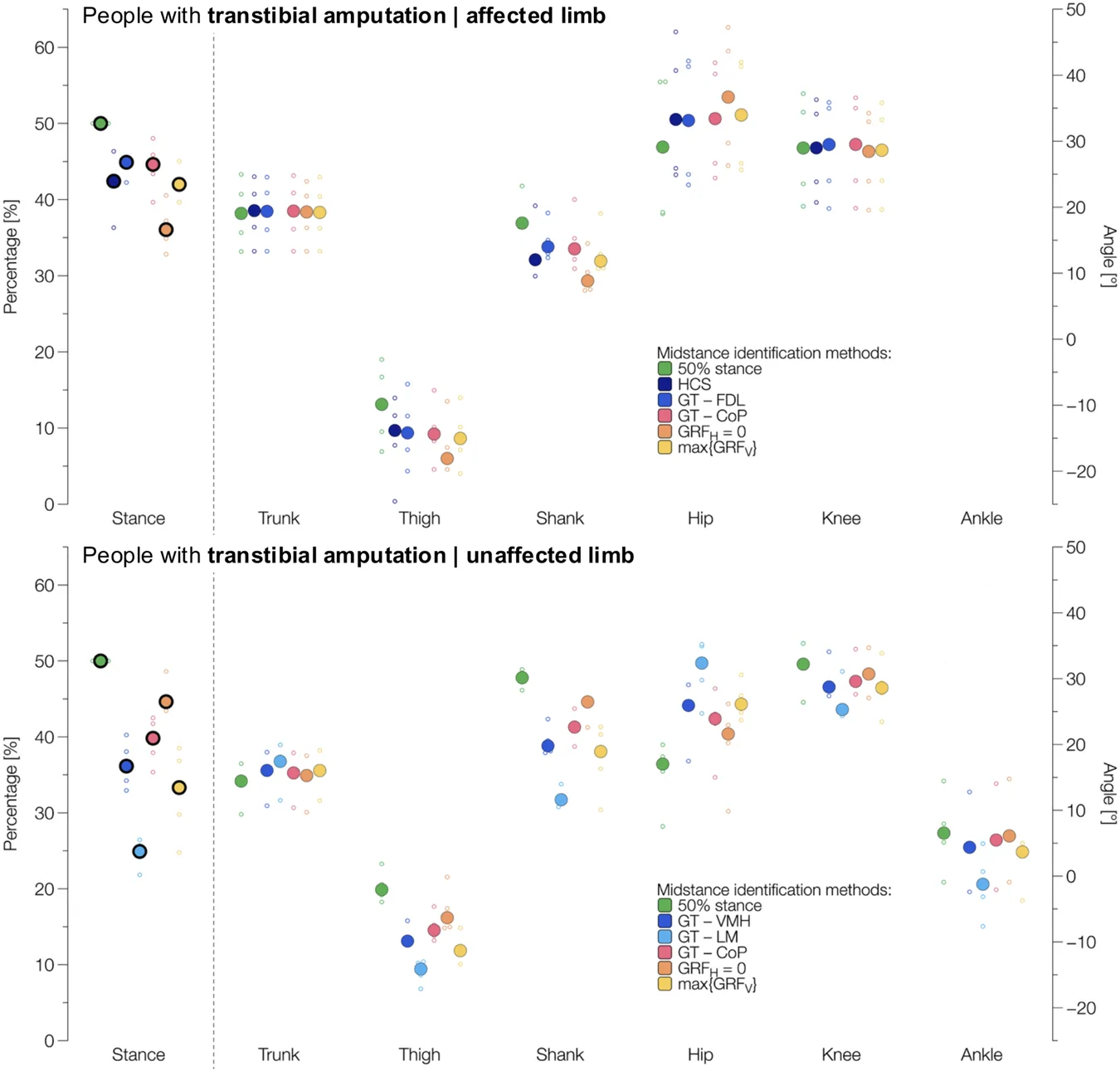
Distribution of kinematic and temporal parameters at midstance in athletes with transtibial amputation. Data are presented according to the median and participants’ distribution. GT–K not applicable for the affected limb, and HCS not applicable for the unaffected limb. Abbreviations: CoP–center of pressure; FDL–lateral marker of the RSP, 5 cm from its tip; GRF–ground reaction force; GT–greater trochanter; H–horizontal (i.e., anterior-posterior); HCS–horizontal contralateral shank; K–prosthetic knee center; LM–lateral malleolus; V–vertical; VMH‐5th metatarsal distal head.

**FIGURE 6:**
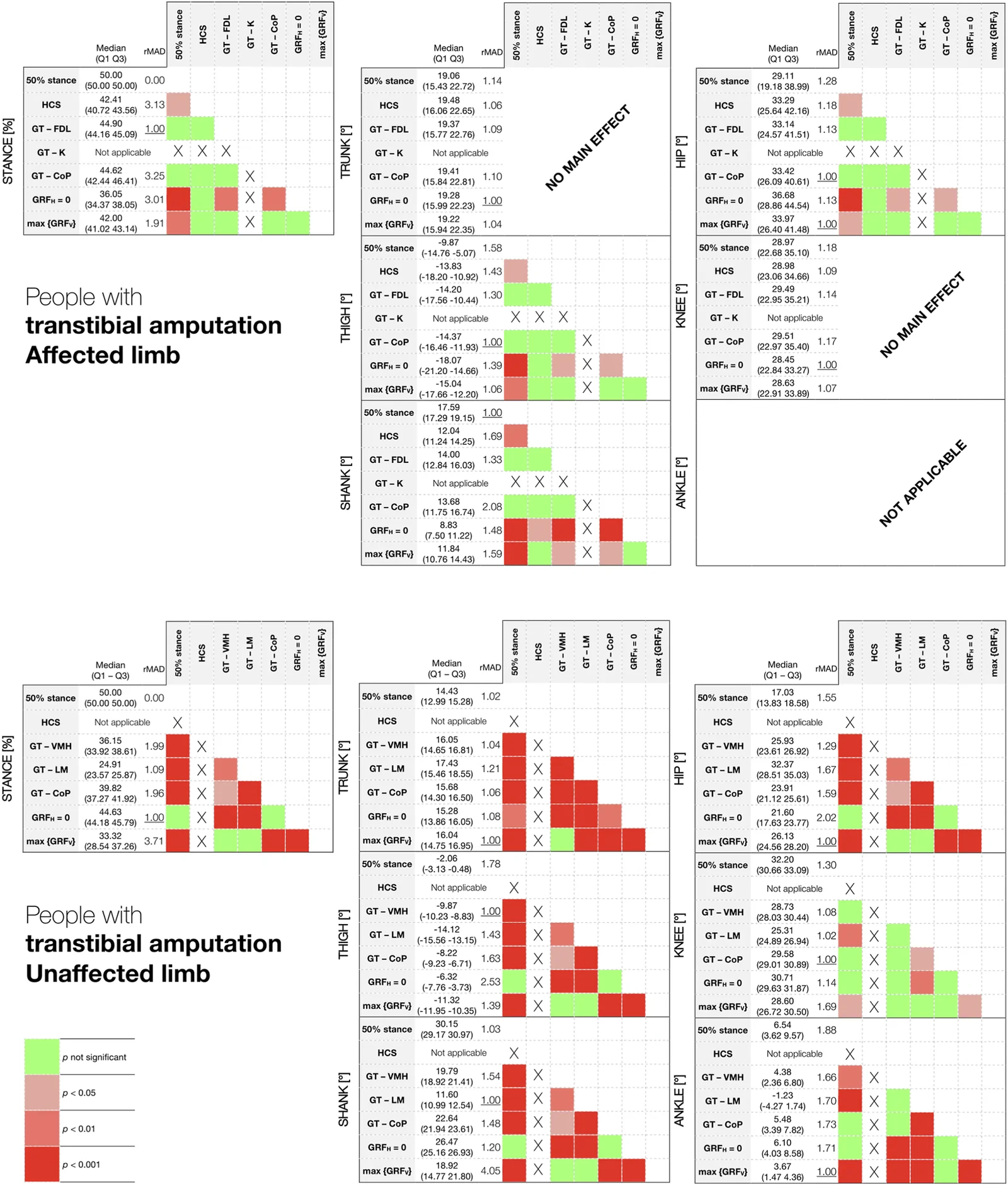
Post-hoc comparison between midstance identification methods in sprinters with transtibial amputation. Data are reported as median (1st quartile 3rd quartile) and relative median absolute deviation. Comparisons between methods are represented by T-intersections in the tables. Statistical significance is represented by the color scale. Abbreviations: CoP–center of pressure; FDL–lateral marker of the RSP, 5 cm from its tip; GRF–ground reaction force; GT–greater trochanter; H–horizontal (i.e., anterior-posterior); HCS–horizontal contralateral shank; K–prosthetic knee center; LM–lateral malleolus; rMAD–relative median absolute deviation; V–vertical; VMH‐5th metatarsal distal head.

**FIGURE 7:**
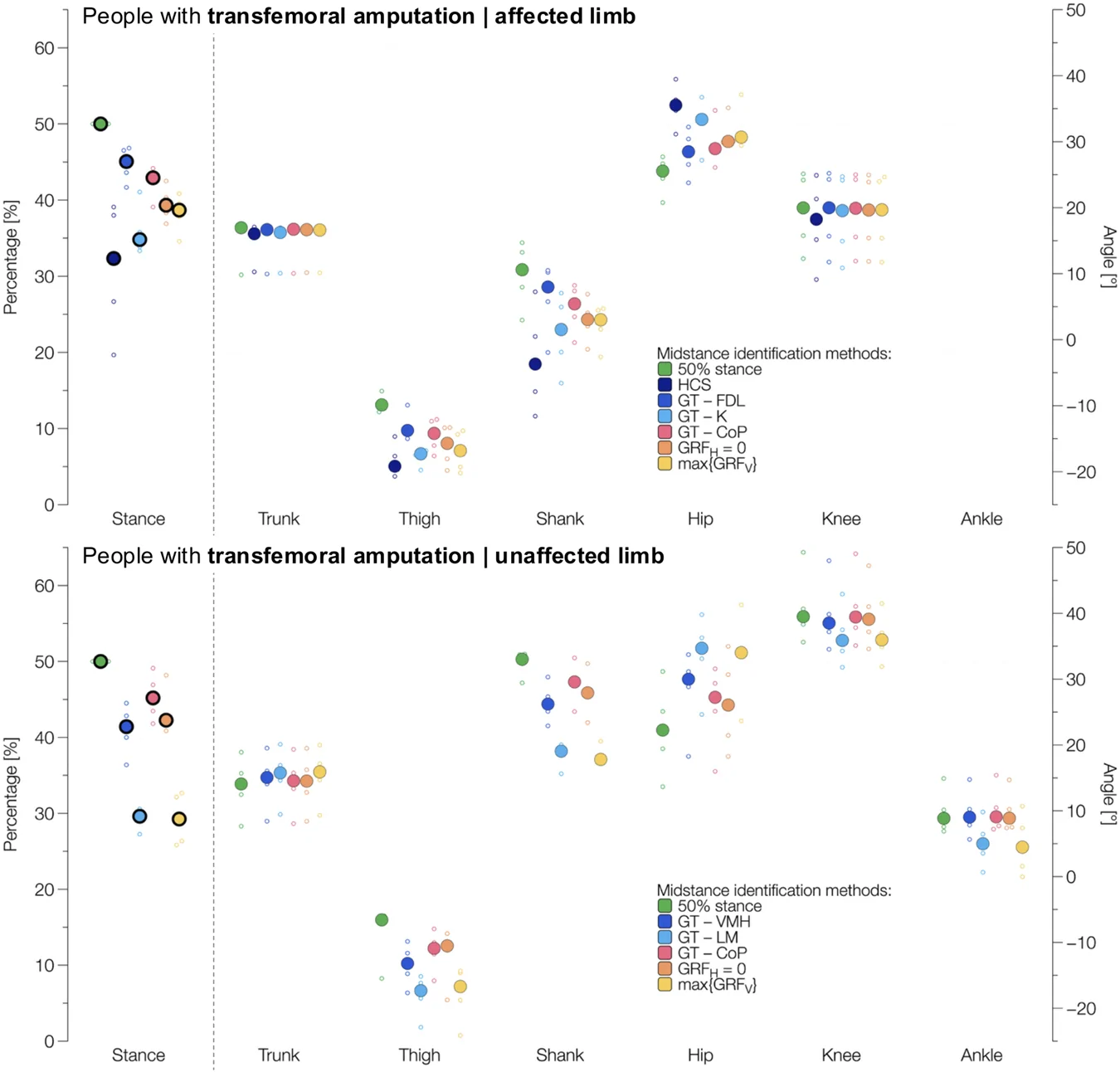
Distribution of kinematic and temporal parameters at midstance in athletes with transfemoral amputation. Data are presented according to the median and participants’ distribution. HCS not applicable for the unaffected limb. Abbreviations: CoP–center of pressure; FDL–lateral marker of the RSP, 5 cm from its tip; GRF–ground reaction force; GT–greater trochanter; H–horizontal (i.e., anterior-posterior); HCS–horizontal contralateral shank; K–prosthetic knee center; LM–lateral malleolus; V–vertical; VMH‐5th metatarsal distal head.

The results are graphically illustrated in Figures 3–8, which present data distribution, intra-method variability, and *post hoc* comparisons for the outcomes described below.

### 3.1 Able-bodied athletes

The one-way repeated measures ANOVA revealed a significant effect across methods (F = 50.59, *p* < 0.001, $\eta_{p}^{2}$ : 0.71) for the percentage of the stance at which the midstance occurred. Differences were also found in all kinematic parameters: trunk (F = 13.21, *p* < 0.001, $\eta_{p}^{2}$ : 0.39), thigh (F = 43.89, *p* < 0.001, $\eta_{p}^{2}$ : 0.68), shank (F = 58.62, *p* < 0.001, $\eta_{p}^{2}$ : 0.74), hip (F = 35.65, *p* < 0.001, $\eta_{p}^{2}$ : 0.63), knee (F = 17.57, *p* < 0.001, $\eta_{p}^{2}$ : 0.46), and ankle (F = 20.24, *p* < 0.001, $\eta_{p}^{2}$ : 0.49) angles. Figures 3, 4 report mean and data distribution, as also rMAD, Bonferroni *post hoc* comparisons, Cohen’s *d*, and ICCs.

### 3.2 Athletes with transtibial amputation

Only the trunk and the knee angle of the AL did not show statistically significant differences between methods. Conversely, all the other parameters exhibited statistically significant differences among methods, specifically: the percentage of stance (X^2^_F_ = 16.08, *p* = 0.007, W: 0.80) as well as thigh (X^2^_F_ = 15.57, *p* = 0.008, W: 0.78), shank (X^2^_F_ = 17.43, *p* = 0.004, W: 0.87), and hip (X^2^_F_ = 15.00, *p* = 0.010, W: 0.75) angles. HCS results were not reported and were excluded from the statistical analysis, as the postural cue required to identify the midstance instant was not observed in two out of four subjects with this method.

Regarding the UL, the percentage of stance was different across methods (X^2^_F_ = 19.00, *p* = 0.002, W: 0.95). Similarly, trunk (X^2^_F_ = 19.57, *p* = 0.002, W: 0.98), thigh (X^2^_F_ = 19.00, *p* = 0.002, W: 0.95), shank (X^2^_F_ = 19.00, *p* = 0.002, W: 0.95), hip (X^2^_F_ = 19.00, *p* = 0.002, W: 0.95), knee (X^2^_F_ = 14.86, *p* = 0.011, W: 0.74), and ankle (X^2^_F_ = 18.00, *p* = 0.003, W: 0.90) angles differed as well. Figure 5 illustrates median and data distribution. Figure 6 reported data, rMAD and the Bonferroni *post hoc* comparisons.

### 3.3 Athletes with transfemoral amputation

Regarding the AL, only the trunk angle was not significantly affected by the identification of midstance instant according to the different methods. By contrast, the percentage of stance (X^2^_F_ = 18.59, *p* = 0.005, W: 0.77), the absolute thigh angle (X^2^_F_ = 19.18, *p* = 0.004, W: 0.80), and the absolute shank angle (X^2^_F_ = 19.18, *p* = 0.004, W: 0.80), as well as the hip (X^2^_F_ = 17.89, *p* = 0.007, W: 0.75) and the knee (X^2^_F_ = 18.64, *p* = 0.005, W: 0.78) angles changed among the different methods.

In the UL, temporal and kinematic parameters differed among methods. The main effects are listed below: percentage of stance (X^2^_F_ = 18.38, *p* = 0.003, W: 0.91), trunk (X^2^_F_ = 18.38, *p* = 0.003, W: 0.91), thigh (X^2^_F_ = 18.38, *p* = 0.003, W: 0.91), and shank (X^2^_F_ = 16.57, *p* = 0.005, W: 0.83) absolute angles, as also hip (X^2^_F_ = 16.57, *p* = 0.005, W: 0.83), knee (X^2^_F_ = 17.51, *p* = 0.004, W: 0.88), and ankle (X^2^_F_ = 15.43, *p* = 0.009, W: 0.77) relative angles. Figure 7 showed median and data distribution, while Figure 8 rMAD and the Bonferroni *post hoc* comparisons.

## 4 Discussion

Midstance represents a critical event in running for both able-bodied athletes and athletes with lower-limb amputation. Altered midstance mechanics can compromise running stability, as the center of mass passes over a reduced base of support (Silva and Stergiou, 2020), and can directly affect prosthetic alignment (Chen et al., 2016; Migliore et al., 2021). Since midstance plays a key role during the stance phase, it is essential to evaluate whether different identification methods correspond to different athlete postures and, consequently, lead to different interpretations of sprinting biomechanics (Inostroza-Ríos et al., 2025). Therefore, trunk angle and lower-limb sagittal and joint angles were computed and reported to directly quantify the practical impact of method selection on the resulting kinematic posture and its biomechanical interpretation.

Among methods requiring force platforms, only GRF_H_ = 0 and max{GRF_V_} are supported by limited evidence in the literature (Richards, 2008; Inostroza-Ríos et al., 2025; Beck et al., 2016). In the absence of force platforms, practitioners may rely on visual or kinematic cues (Fellin et al., 2010), although they are strongly operator dependent. This coexistence of kinetic-based and visually-based methods raises a critical issue regarding their consistency and interchangeability. This concern has also been highlighted in previous studies showing that kinetic transitions and kinematic postures do not necessarily coincide during sprinting (Ciacci et al., 2010). Indeed, the use of different criteria among athletes, coaches, and clinicians may result in the identification of different instants within the stance phase, thereby generating inconsistencies in both kinematic and kinetic outcomes.

The main findings indicate that, in able-bodied athletes and in the unaffected limb of athletes with lower-limb amputation, kinetic-based methods differ from each other, as they identified midstance at different temporal instants. Conversely, for the prosthetic limb of both PTTA and PTFA groups, the three kinetic-based methods showed greater consistency in detecting midstance (Figures 5–8). This difference between the two limbs in athletes with lower-limb amputation may reflect the altered ground reaction force patterns of the affected limb, typically characterized by reduced vertical GRF and modified distribution of braking and propulsive impulses (Grabowski et al., 2010; Sakata et al., 2024).

These findings have important methodological implications. For able-bodied athletes and for the unaffected limb, consistency in the selection of the kinetic-based method is essential. In contrast, for the affected limb, there seems to be greater flexibility, provided that the same method is applied consistently across repeated assessments.

Kinematic methods showed limited consistency, producing significantly different kinematic configurations. Thus, these methods identify distinct biomechanical events rather than a common midstance instant. This is consistent with previous studies showing that the accuracy of kinematic event detection depends on the selected method (Fellin et al., 2010; Alvim et al., 2015; Smith et al., 2015). Notably, validation of kinematic detection remains poorly investigated in individuals with lower-limb amputation and cannot be assumed to transfer directly from able-bodied population (Aftab and Ahmed, 2022). Moreover, the agreement of kinematic methods with kinetic-based methods was only partial and dependent on both the method and the athlete group.

Body posture captured at the theoretical midpoint of the stance phase (50%) showed differences when compared with kinetic-based methods in the able-bodied group. In athletes with lower-limb amputation, those differences depended on limb and level of amputation, similarly to differences obtained with GRF_H_ = 0 or GT–CoP methods. These findings indicate that the 50% stance instant may capture a group-specific body posture, which does not necessarily represent a key biomechanical transition. Notably, all other methods identified midstance earlier than 50% of stance phase (Figures 3, 5, 7). Although the 50% of stance is not indicative of any conventionally defined midstance, the MAD calculated relative to this temporal reference provides an indicative reference for quantifying the temporal offset among midstance instants identified across different methods. It is worthy to mark that the use of 50% stance may introduce systematic temporal bias and lead to inconsistent biomechanical interpretation.

The GT–VMH method was partially aligned with the kinetic-based methods in the able-bodied group (Figure 4). In PTTA and for the UL, it aligned with max{GRF_V_} method but differed from GRF_H_ = 0, whereas an opposite trend was observed in PTFA. This suggests that its validity may depend on the level of amputation. The lower variability observed in the PTTA (i.e., rMAD <1.99) compared to PTFA (i.e., rMAD up to 3.18) indicates that this method may be more suitable for athletes with transtibial amputation. A similar behavior was observed for the GT–FDL method in the affected limb, supporting its use as a surrogate for GT–VMH. Conversely, for PTFA, the GT–K method emerged as a more robust alternative, showing lower variability and consistency with both GRF_H_ = 0 and max{GRF_V_} methods.

The GT–LM method showed significant differences with all kinetic-based methods in the AB group and identified midstance consistently earlier than all other methods (15.8%–30.6% of stance). This resulted in substantially different kinematics, limiting its suitability for midstance detection despite it was not significantly different from max{GRF_V_} when applied to the UL of both PTTA and PTFA.

The HCS method did not differ from GRF_H_ = 0 and GT–CoP in the AB group and it was also comparable to both GRF_H_ = 0 and max{GRF_V_} in PTTA and PTFA groups. However, it exhibited the highest variability masking any statistical difference (Figures 4, 6, 8). This variability may be attributed to the occurrence of a visually defined postural cue by the operator (i.e., contralateral shank parallel to the ground), which could affect the robustness and generalizability of the method. Furthermore, the applicability of the HCS method proved to be limited across the PTTA group: in two out of four athletes the postural cue did not occur during the UL stance. This limitation likely reflects the asymmetric mechanics of sprinters with unilateral transtibial amputation compared to able-bodied sprinters (Emonds and Mombaur, 2021). Therefore, the inability to consistently identify the target posture highlights the limited feasibility of the method and raises concerns regarding its applicability. Despite its simplicity, the HCS method appears to lack robustness and may lead to non-reproducible kinematics outcomes.

Overall, and limited to the AB group where ICC analyses were feasible, the consistency among methods can be considered generally acceptable for trunk and knee angles. In contrast, consistency was moderate for ankle angle, and poor for stance percentage as well as for thigh, shank, and hip angles. In these latter cases, the choice of the method appears to be particularly critical, as it can substantially influence the resulting estimates and therefore the interpretation of the kinematic outcomes.

The present findings should be interpreted in light of some methodological limitations. First, the study included only high-level competitive sprinters. Therefore, the results may not be generalized to recreational sprinters or long-distance runners. Moreover, the marker-based model used to compute joint and segmental kinematics was not subjected to a formal reliability analysis, limiting the possibility of quantifying its measurement precision. Additionally, the difference in sample sizes of PTTA and PTFA groups compared to AB group precluded the use of complementary agreement analyses. It should be noted that ICC evaluates relative consistency among methods and does not fully replace absolute agreement analyses, such as Bland–Altman analysis. Consequently, the present findings should be considered exploratory and interpreted as primary guidance, rather than definitive benchmarks.

In conclusion, midstance can be identified using either kinetic or kinematic approaches. The tested methods showed substantial differences in both temporal identification and the resulting body posture, indicating that they should not be used interchangeably. Consistent use of a single method is therefore essential to ensure reliable data interpretation. Furthermore, since the performance of each method may vary across athlete populations, its choice should be based on the population under investigation. From a practical perspective, for coaches and practitioners who primarily rely on kinematic methods, this exploratory study suggests that GT–VMH method appears most suitable for able-bodied athletes and unaffected limbs of PTTA and PTFA athletes, whereas GT–FDL and GT–K methods may be more appropriate for the affected limb of PTTA and PTFA athletes, respectively. In contrast, the HCS and GT–LM should be used with caution because of their high variability or systematic temporal bias.

## Data Availability

The raw data supporting the conclusions of this article will be made available by the authors, without undue reservation.

## References

[B1] Aftab Z. Ahmed G. (2022). Validity of dual-minima algorithm for heel-strike and toe-off prediction for the amputee population. Prosthesis 4 (2), 224–233. 10.3390/prosthesis4020022

[B2] Alvim F. Cerqueira L. Netto A. D. ’A. Leite G. Muniz A. (2015). Comparison of five kinematic-based identification methods of foot contact events during treadmill walking and running at different speeds. J. Appl. Biomechanics 31 (5), 383–388. 10.1123/jab.2014-0178 25950421

[B3] Arendse R. E. Noakes T. D. Azevedo L. B. Romanov N. Schwellnus M. P. Fletcher G. (2004). Reduced eccentric loading of the knee with the pose running method. Med. Sci. Sports Exerc. 36 (2), 272–277. 10.1249/01.MSS.0000113684.61351.B0 14767250

[B4] Armand S. Sawacha Z. Goudriaan M. Horsak B. van der Krogt M. Huenaerts C. (2024). Current practices in clinical gait analysis in Europe: a comprehensive survey-based study from the european society for movement analysis in adults and children (ESMAC) standard initiative. Gait and Posture 111 (June), 65–74. 10.1016/j.gaitpost.2024.04.014 38653178

[B5] Beck O. N. Taboga P. Grabowski A. M. (2016). Characterizing the mechanical properties of running-specific prostheses. PLoS ONE 11 (12), e0168298. 10.1371/journal.pone.0168298 27973573 PMC5156386

[B6] Blickhan R. (1989). The spring-mass model for running and hopping. J. Biomechanics 22 (11–12), 1217–1227. 10.1016/0021-9290(89)90224-8 2625422

[B7] Bramah C. Tawiah-Dodoo J. Rhodes S. Elliott J. D. Dos’Santos T. (2024). The sprint mechanics assessment score: a qualitative screening tool for the In-Field assessment of sprint running mechanics. Am. J. Sports Med. 52 (6), 1608–1616. 10.1177/03635465241235525 38544464 PMC11064464

[B8] Chen C. W. J. Heim W. Fairley K. Clement R. J. Biddiss E. Torres-Moreno R. (2016). Evaluation of an instrument-assisted dynamic prosthetic alignment technique for individuals with transtibial amputation. Prosthetics Orthot. Int. 40 (4), 475–483. 10.1177/0309364615574161 25762611

[B9] Ciacci S. Michele R. D. Franco M. (2010). Kinematic analysis of the braking and propulsion phases during the support time in sprint running. Gait and Posture 31 (2), 209–212. 10.1016/j.gaitpost.2009.10.007 19926284

[B10] Di Marco R. Breban S. G. Zullo G. Gariboldi F. Scapinello M. Migliore G. L. (2025). A motion capture protocol for the kinematic analysis of transfemoral and transtibial sprinters. Front. Bioeng. Biotechnol. 13, 1655295. 10.3389/fbioe.2025.1655295 41262427 PMC12623396

[B11] Dugan S. A. Krishna P. B. (2005). Biomechanics and analysis of running gait. Phys. Med. Rehabilitation Clin. N. Am. 16 (3), 603–621. 10.1016/j.pmr.2005.02.007 16005396

[B12] Emonds A. L. Mombaur K. (2021). Asymmetry in three-dimensional sprinting with and without running-specific prostheses. Symmetry 13 (4), 580. 10.3390/sym13040580

[B13] Fellin R. E. Rose W. C. Royer T. D. Davis I. S. (2010). Comparison of methods for kinematic identification of footstrike and toe-off during overground and treadmill running. J. Sci. Med. Sport 13 (6), 646–650. 10.1016/j.jsams.2010.03.006 20478742 PMC3266867

[B14] Fletcher G. Bartlett R. Romanov N. Ali F. (2008). Pose® method technique improves running performance without economy changes. Int. J. Sports Sci. and Coach. 3 (April), 365–380. 10.1260/174795408786238506

[B15] Grabowski A. M. McGowan C. P. McDermott W. J. Beale M. T. Kram R. Herr H. M. (2010). Running-specific prostheses limit ground-force during sprinting. Biol. Lett. 6 (2), 201–204. 10.1098/rsbl.2009.0729 19889694 PMC2865064

[B16] Hadj-Moussa F. Ngan C. C. Andrysek J. (2022). Biomechanical factors affecting individuals with lower limb amputations running using running-specific prostheses: a systematic review. Gait Posture 92, 83–95. 10.1016/j.gaitpost.2021.10.044 34837772

[B17] Inostroza-Ríos F. Merino-Muñoz P. Sánchez-Ramírez C. Garrido A. B. Pérez-Contreras J. Cancino-Jimenez J. (2025). The temporal structure of the running cycle, an essential element in the analysis: a critical review. Biomechanics 5 (2), 40. 10.3390/biomechanics5020040

[B18] Kapri E. Mehta M. Kiran S. (2021). Biomechanics of running: an overview on gait cycle. Int. J. Of Phys. Educ. Fit. And Sports 10 (3), 1–9. 10.34256/ijpefs2131

[B19] Koo T. K. Li M. Y. (2016). A guideline of selecting and reporting intraclass correlation coefficients for reliability research. J. Chiropr. Med. 15 (2), 155–163. 10.1016/j.jcm.2016.02.012 27330520 PMC4913118

[B20] Lath F. Koopmann T. Faber I. Baker J. Schorer J. (2021). Focusing on the coach’s eye; towards a working model of coach decision-making in talent selection. Psychol. Sport Exerc. 56, 102011. 10.1016/j.psychsport.2021.102011

[B21] Migliore G. L. Petrone N. Hobara H. Nagahara R. Miyashiro K. Costa G. F. (2021). Innovative alignment of sprinting prostheses for persons with transfemoral amputation: exploratory study on a gold medal paralympic athlete. Prosthetics Orthot. Int. 45 (1), 46–53. 10.1177/0309364620946910 33834744

[B22] Ounpuu S. (1990). The biomechanics of running: a kinematic and kinetic analysis. Instr. Course Lect. 39, 305–318. 2335745

[B23] Perry J. Burnfield J. (2010). Gait Analysis: Normal and Pathological Function. 2nd ed. Boca Raton, FL: CRC Press. 10.1201/9781003525592

[B24] Richards J. (2008). Biomechanics in Clinic and Research. Edinburgh, United Kingdom: Churchill Livingstone/Elsevier.

[B25] Romanov N. (2002). Dr. Romanov’s Pose Method of Running. Coral Gables, FL: Pose Tech. Corp.

[B26] Romanov N. Fletcher G. (2007). Runners do not push off the ground but fall forwards *via* a gravitational torque. Sports Biomech. 6 (3), 434–452. 10.1080/14763140701491625 17933203

[B27] Sakata H. Hashizume S. Amma R. Hisano G. Murata H. Takemura H. (2024). Anterior-posterior ground reaction forces across a range of running speeds in unilateral transfemoral amputees. Sports Biomech. 23 (1), 69–80. 10.1080/14763141.2020.1822434 33112726

[B28] Shrout P. E. Fleiss J. L. (1979). Intraclass correlations: uses in assessing rater reliability. Psychol. Bull. 86 (2), 420–428. 10.1037/0033-2909.86.2.420 18839484

[B29] Silva L. M. Stergiou N. (2020). “Chapter 7 - the basics of gait analysis”, in Biomechanics and Gait Analysis. Editor Stergiou N. (Cambridge, Massachusetts, United States: Academic Press). 10.1016/B978-0-12-813372-9.00007-5

[B30] Smith L. Preece S. Mason D. Bramah C. (2015). A comparison of kinematic algorithms to estimate gait events during overground running. Gait and Posture 41 (1), 39–43. 10.1016/j.gaitpost.2014.08.009 25212739

[B31] Souza R. B. (2016). An evidence-based videotaped running biomechanics analysis. Phys. Med. Rehabilitation Clin. N. Am. 27 (1), 217–236. 10.1016/j.pmr.2015.08.006 26616185 PMC4714754

[B32] Thordarson D. B. (1997). RUNNING BIOMECHANICS. Clin. Sports Med. 16 (2), 239–247. 10.1016/S0278-5919(05)70019-3 9238307

[B33] Wei R. X. Au I. P. H. Lau F. O. Y. Zhang J. H. Chan Z. Y. S. MacPhail A. J. C. (2021). Running biomechanics before and after pose® method gait retraining in distance runners. Sports Biomech. 20 (8), 958–973. 10.1080/14763141.2019.1624812 31364959

[B34] Wu Ge Cavanagh P. R. (1995). ISB recommendations for standardization in the reporting of kinematic data. J. Biomechanics 28 (10), 1257–1261. 10.1016/0021-9290(95)00017-C 8550644

[B35] Wu G. Siegler S. Allard P. Kirtley C. Leardini A. Rosenbaum D. (2002). ISB recommendation on definitions of joint coordinate system of various joints for the reporting of human joint motion - part I: ankle, hip, and spine. J. Biomechanics 35 (4), 543–548. 10.1016/S0021-9290(01)00222-6 11934426

[B36] Wu Ge Van Der Helm F. C. T. Veeger H. E. J. Makhsous M. Van Roy P. Anglin C. (2005). ISB recommendation on definitions of joint coordinate systems of various joints for the reporting of human joint motion - part II: shoulder, elbow, wrist and hand. J. Biomechanics 38 (5), 981–992. 10.1016/j.jbiomech.2004.05.042 15844264

